# Poisoning by Strychnia—Recovery

**Published:** 1867-03

**Authors:** Julien S. Sherman

**Affiliations:** Chicago


### ARTICLE XV.

### POISONING BY STRYCHNIA.—RECOVERY.

By JULIEN S. SHERMAN, M.D., Chicago.

The frequent fatal results of poisonous amounts of strychnia taken into the stomach, render the treatment of those which recover of considerable interest. The following case occurred on February 7th:—

Charles W., a resident of Chicago, *æt.* 23 years, of more than usual muscular development, desiring to "shuffle off this mortal coil," procured two grains of strychnia at 9 o'clock in the morning, and immediately deposited the same in his stomach. I was called to see him at 10 o'clock, and learned that he had suddenly fallen to the floor, complaining of inability to move his limbs, while his whole muscular system was in a state of tremor. The characteristic symptoms of poisoning from strychnia were now present. The spasms were frequent, and the rigidity of the cervical muscles marked. He complained of a sense of constriction in the fauces, and at times could only with difficulty swallow. The extremities were cool, and the mouth filled with frothy saliva. Pulse 115 to the minute; countenance extremely anxious.

An emetic of sulphate of zinc and ipecac. was immediately given and soon repeated, when free emesis was produced. A mixture of equal parts of tinct. cannabis indica and tinct. of camphor was given in drachm doses every 10 minutes; chloroform, by inhalation, was used for the first half-hour, when it was omitted, and the mixture alone given.

The convulsions, at first, were as frequent as once every 10 minutes, and similar to the action produced by an electric shock. At 11½ o'clock, they were greatly reduced in frequency, and the remedy was given at longer intervals. The symptoms soon became worse, and the dose was increased. They were again controlled, and gradually ceased entirely. By 11 o'clock at night, the patient was sleeping, and the next morning complained of extreme exhaustion, with general muscular soreness. Tonics and a nutritious diet soon completed his recovery.

During the 12 hours of treatment, nearly 4 ounces of the mixture was given, and yet none of the symptoms of intoxication from cannabis indica were developed.

